# CBX8 exhibits oncogenic properties and serves as a prognostic factor in hepatocellular carcinoma

**DOI:** 10.1038/s41419-018-1288-0

**Published:** 2019-01-18

**Authors:** Bo Tang, Yu Tian, Yong Liao, Zeming Li, Shuiping Yu, Huizhao Su, Fudi Zhong, Guandou Yuan, Yan Wang, Hongping Yu, Stephen Tomlinson, Xiaoqiang Qiu, Songqing He

**Affiliations:** 1 Department of Hepatobiliary Surgery, The First Affiliated Hospital of Guangxi Medical University, Nanning, Guangxi China; 2 CAS Key Laboratory of Separation Sciences for Analytical Chemistry, Dalian Institute of Chemical Physics, Chinese Academy of Sciences, Dalian, China; 3 Department of Vascular Surgery, The Second Hospital of Dalian Medical University, Dalian, Liaoning China; 4 Department of Microbiology and Immunology, Medical University of South Carolina, Charleston, SC USA; 5 School of Public Health, Guangxi Medical University, Nanning, Guangxi China

## Abstract

Polycomb group family is a class of proteins that have important roles in both physiological and pathological processes, and its family member Chromobox homolog 8 (CBX8) regulates cell differentiation, aging, and cell cycle progression in numerous carcinomas; however, the effects and underlying mechanisms of CBX8 in hepatocellular carcinoma (HCC) are rarely reported. We found that CBX8 expression in clinical HCC specimens correlates inversely with patient survival. In HCC cells, we found that enforced overexpression of CBX8 induces epithelial–mesenchymal transition, invasive migration, and stem cell-like traits, which are associated with increased tumor growth and metastasis in mice. Conversely, CBX8 silencing inhibits the aggressive phenotype of HCC cells that have high CBX8 expression. Mechanistically, CBX8 modulates H3K27me3 in the gene promoter of bone morphogenetic protein 4 (BMP4), which is associated with active BMP4 transcription and, consequently, the activation of Smads and mitogen-activated protein kinases. BMP4 expression reverses the effects of CBX8 silencing in inhibiting epithelial–mesenchymal transition, stemness, and metastasis. Our results establish CBX8 as a critical driver of HCC stem cell-like and metastatic behaviors and characterize its role in modulating BMP4 expression. These findings have implications for the targeting of CBX8 as an approach to HCC prognosis and treatment.

## Highlights

1. CBX8 expression in clinical HCC specimens correlates inversely with patient survival.

2. CBX8 promotes HCC proliferation, mobility and invasion (epithelial–mesenchymal transition, EMT), and stemness.

3. CBX8 modulates H3K27me3 in the gene promoter of BMP4, which is associated with active BMP4 transcription, and consequently activated Smads and MAP kinases.

## Introduction

Hepatocellular carcinoma (HCC) is one of the leading causes of cancer-related death and the fifth most common cancer in the world. About 500,000–1,000,000 new cases occur each year, more than 50% of which occur in China^1^. Although surgery, transcatheter arterial chemical embolism, radiofrequency ablation, and transplantation have been widely applied in clinical treatment, patients with HCC still have poor prognosis because of the insidious onset, high malignancy, high invasiveness, rapid progression, and high recurrence rate of HCC^2,3^. Moreover, markers used for HCC prognosis prediction after resection are not satisfactory due to their poor accuracy and reproducibility. Therefore, it is important to explore novel markers to improve HCC diagnosis and treatment.

The polycomb group proteins, first discovered in Drosophila, are essential regulators of cell proliferation and differentiation, which are often deregulated in human cancers and contribute to the development of cancer^4,5^. Polycomb proteins are mainly comprised of two complexes, polycomb repressive complex 1 and 2 (PRC1 and PRC2), whose functions are to maintain transcriptional repression. Chromobox homolog 8 (CBX8), a homolog of the Drosophila polycomb protein, is a component of PRC1, which has been shown to have a critical role in the pathogenesis of cancer. As a transcriptional repressor, CBX8 regulates numerous target genes that are important for cell growth and survival, including the tumor suppressor gene INK4a/ARF locus^6^, which is involved in cell-fate decisions, and AF9, which is implicated in the development of acute leukemia^7^. Recent studies have revealed that DNA damage induces CBX8 upregulation, and CBX8 knockdown results in more severe DNA damage, indicating that CBX8 is a key regulator of DNA repair. CBX8 is upregulated in human esophageal carcinoma and participates in DNA repair to promote esophageal carcinogenesis^8^. CBX8 is also upregulated in colorectal cancer, and CBX8 overexpression indicates poor prognosis^9^. Although evidence suggests that CBX8 expression is correlated with the tumor generation and development, few studies have focused on the function and mechanism of CBX8 in HCC.

Migration and invasion are important malignant biological behaviors of HCC. Increasing evidence indicates that epithelial–mesenchymal transition (EMT) is one of the key initiation steps in metastasis. EMT is characterized by increased epithelial-like molecules, decreased mesenchymal-like markers, and loss of cellular polarity and junctions^10^. The progression of EMT stimulates cancer cell motility, migration, and invasion properties and has been regarded as an early indicator of metastasis^11^. Therefore, clarifying the mechanism of EMT will help us to understand how HCC metastasizes.

In this study, we determined that CBX8 expression in HCC tissues is inversely correlated with patient survival. The overexpression of CBX8 in HCC cells induces EMT, migration, invasion, and stem cell-like traits in vitro and enhances the cancer stem cell-like and metastatic capacity in vivo. Conversely, silencing of CBX8 in HCC cells inhibits these processes. These functional effects of CBX8 are exerted by its ability to control bone morphogenetic protein 4 (BMP4) transcriptional expression via H3K27me3, and to modulate Smads and the mitogen-activated protein kinase (MAPK) signaling pathway. Our findings provide novel mechanistic insight into the role of CBX8 in HCC metastasis, and imply that the enzymatic activity of CBX8 may be a therapeutic target for HCC.

## Materials and methods

### Human tissue specimens and cell lines

A total of 153 paired, paraffin-embedded primary specimens were obtained from patients undergoing HCC surgery without radiotherapy or chemotherapy. The patients were diagnosed according to their clinical pathological characteristics at the Affiliated Hospital of Guilin Medical University from 2010 to 2014. Eighty-three pairs of fresh HCC tissues and corresponding adjacent non-tumor tissues obtained from the Affiliated Hospital of Guilin Medical University were stored at −80 °C immediately after surgery and were used for western blotting. Informed consent was obtained from all patients, and the study was approved by the Ethics Committee of Guilin Medical University.

Human hepatocytes (L02) and hepatic tumor cell lines (SMMC-7721, HepG2, Huh7, and Hep3B) were purchased from the Cell Bank, Chinese Academy of Sciences, Shanghai, China. The cells were cultured in RPMI-1640, 10% fetal bovine serum (FBS) (Gibco), 1% penicillin, and streptomycin.

### Quantitative reverse transcription-PCR

Total RNA was extracted using Trizol reagent (Invitrogen), and complementary DNA (cDNA) was synthesized using SuperScript II Reverse Transcriptase (Invitrogen). Quantitative reverse transcription-PCR (qRT-PCR) and data collection were performed with an ABI PRISM 7900HT sequence detection system.

### Western blotting

Cells or tissues were lysed in ice-cold radioimmunoprecipitation assay buffer containing protease inhibitors. After centrifugation at 12,000 rpm for 20 min at 4 °C, the lysates were separated by sodium dodecyl sulfate-polyacrylamide gel electrophoresis and transferred to polyvinylidene difluoride membranes for western blotting using ECL detection reagents.

### Immunohistochemistry

Immunohistochemistry (IHC) staining for CBX8 was performed in 153 paired paraffin-embedded HCC tissue specimens. Briefly, after baking and de-paraffinizing, the tissue sections were stained with anti-CBX8 antibody followed by secondary anti-rabbit horse radish peroxidase-conjugated antibody. CBX8 expression was evaluated blindly by two professional pathologists. The IHC staining score was calculated according to the percentage of positive tumor cells and the staining intensity. The percentage of positive cells was calculated in five views that were randomly selected under microscopy at ×400 magnification. The colored cells accounting for ≤5% were given 0 points, 6–25% 1 point, 26–50% 2 points, 51–75% 3 points, and >75% 4 points. The staining intensity was scored as follows: nuclei without color were given 0 points, with a light brown color 1 point, brown 2 points, and dark brown 3 points. Finally, the two scores were multiplied and the samples were designated as the low expression group (IHC staining score of <4) and high expression group (IHC staining score of ≥4).

### Establishment of CBX8 and BMP4 stable expression and CBX8-knockdown cell lines

pBabe.puro retroviral constructs containing human CBX8 and BMP4 cDNAs and pSuper.retro.puro retroviral constructs containing short hairpin RNA (shRNA) against human CBX8 were prepared by GenePharma, Shanghai, China (shCBX8#1, 5′-GCAUGGAAUACCUCGUGAATT-3′; shCBX8#2, 5′-CGAGUUUCGAAGUGACUCATT-3′; shCBX8#3, 5′-CUUCGAAACAUGGGUUUGUTT-3′). The generation of retrovirus supernatants and transfection of cancer cells were conducted as described previously^12^. Infected cells were selected by adding 2 μg/ml puromycin to the culture medium for 48 h and then maintained in complete medium with 0.5 μg/ml puromycin. Empty retroviral-infected stable cell lines were also produced as described above, except that infected cells were selected by adding 400 μg/ml of G418.

### Cell invasion and motility assays

Invasion of cells was measured in Matrigel (BD)-coated transwell inserts (Costar) containing polycarbonate filters with 8-μm pores as detailed previously^13^. The inserts were coated with 50 μl of 1 mg/ml Matrigel matrix according to the manufacturer’s recommendations. Cells (2 × 10^5^) in 200 μl of serum-free medium were plated in the upper chambers, and 600 μl of medium with 10% FBS was added to the lower wells. After 24 h incubation, cells that migrated to the lower surface of the membrane were quantified following fixing and staining. For each membrane, five random fields were counted. The data are presented as mean ± SD from three independent experiments performed in triplicate. Motility assays were similar to the Matrigel invasion assays, except that the transwell insert was not coated with Matrigel.

### Chromatin immunoprecipitation-seq and ChIP-PCR

Chromatin immunoprecipitation (ChIP) kits were purchased from Millipore, and ChIP experiments were carried out as described^14^. Immunoprecipitated DNA was analyzed on an ABI PRISM 7900HT sequence detection system.

### Hoechst 33342 staining, flow cytometry analysis, and sorting of side population cells

Cells were washed with phosphate-buffered saline, detached from the culture dishes with trypsin and EDTA, pelleted by centrifugation, and resuspended in 37 °C Dulbecco’s modified Eagle’s medium containing 2% FBS at 1 × 10^6^ cells/ml. Cell staining was performed as described^15^. The cells were incubated with Hoechst 33342 (Sigma) at 5 μg/ml either alone or in combination with the ABC transporter inhibitor verapamil (50 μM, Sigma) for 90 min at 37 °C. After staining, the cells were centrifuged and resuspended in Hank’s balanced salt solution (Invitrogen) containing 1 μg/ml propidium iodide and maintained at 4 °C for flow cytometric analysis and sorting. Cell analysis and sorting were performed on a MoFlo cytometer equipped with a Coherent Enterprise II laser-emitting MLUV at 351- and blue 488-nm lines. The Hoechst 33342 emission was first split using a 610-nm dichroic short-pass filter, and red and the blue emissions were collected through 670/30- and 450/65-nm bandpass filters, respectively.

### Holoclonal and clonogenic, and sphere-forming assays

For the holoclone assay, we plated cells at 100 cells/well in a 6-well dish, counted the number of holoclones several days later, and from the percentage of cells that established a holoclone, determined cloning efficiency. For the clonogenic assay, we plated cells at 1000 cells/well in Matrigel or methylcellulose at a 1:1 ratio in 100–200 μl and enumerated colonies 1–2 weeks after plating. Mammosphere culture was performed as described by Dontu et al.^16^ with slight modifications. Single-cell suspensions were plated in ultralow attachment 96-well plates (Costar) at different densities of viable cells. Cells were grown in serum-free epithelial growth medium, supplemented with 1:50 B27 (Invitrogen), 20 ng/ml epidermal growth factor, 20 ng/ml basic fibroblast growth factor (BD), and 10 μg/ml heparin (Sigma). The number of spheroids was counted after 7–10 days.

### Animal experimentation

BABL/c nude mice (4 weeks old) were obtained from the Animal Center of Guangxi Medical University (Nanning, China). All animal experiments were performed according to the National Institutes of Health Animal Use Guidelines on the Use of Experimental Animals. To assess in vivo tumor growth, 2 × 10^6^ cells were injected subcutaneously into each mouse and tumors were measured weekly. Tumor volume was calculated according to the formula length × width^2^/2. After 4 weeks, tumors were removed and weighed. To establish the lung metastasis model, 1 × 10^6^ cells were implanted into nude mice via tail vein injection. After 6 weeks, the lungs were obtained and fixed in 4% paraformaldehyde. The specimen of tumor tissue was embedded in paraffin and routinely stained with hemtoxylin and eosin. Histopathological analysis was performed with a light microscope, and the number of metastatic lung nodules was calculated.

### Microarray analysis

Total RNA of CBX8-knockdown and control cells was extracted for microarray analysis by Genechem. The data were initially normalized by robust multiarray average (RMA) normalization algorithms in expression console software (Affymetrix). Significantly altered genes between CBX8-knockdown cells and control cells were analyzed by scatter plots and the genes up- and downregulated ≥5-fold. Heat maps were visualized using the Java TreeView v1.1.4r3 software. Gene set enrichment analysis was carried out using ConceptGen (http://conceptgen.ncibi.org/core/conceptGen/index.jsp). Gene sets were either obtained from the ConceptGen or from published gene signatures.

### Statistical analysis

Data were recorded as means ± SD. The association between BMP4 and CBX8 expression in HCC tissue was assessed using the Spearman’s rank correlation test. Comparisons between different groups were undertaken using the Student’s *t* test. The limit of statistical significance was *p* < 0.05. Statistical analysis was performed with the SPSS 22.0 software.

## Results

### CBX8 is highly expressed in HCC, and its expression is positively correlated with distant metastasis, and inversely correlated with overall survival

**Fig. 1:**
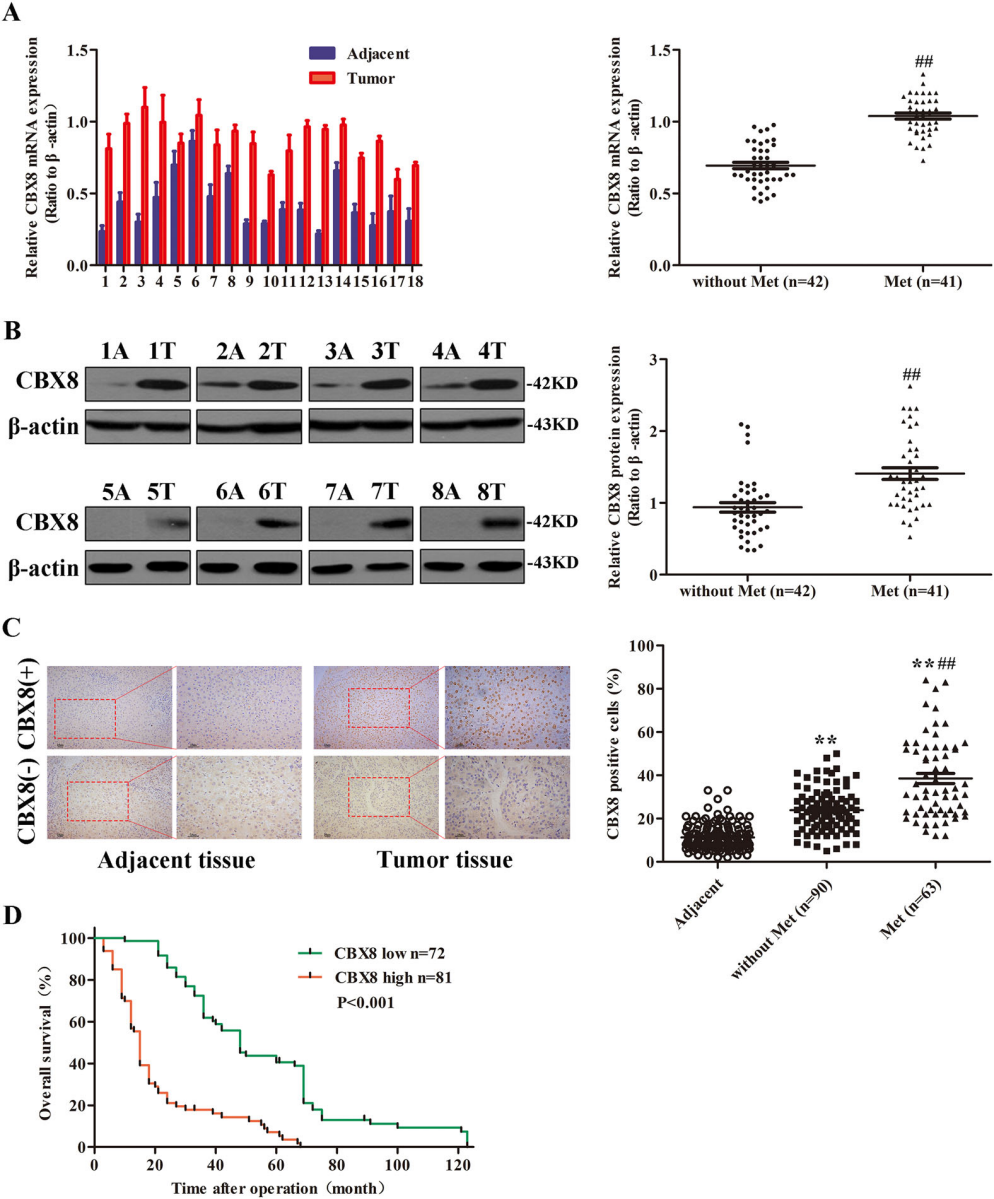
CBX8 is highly expressed in HCCs, and the expression correlates with distant metastasis and patient survival. **a** The expression of CBX8 in HCC and adjacent normal liver tissues of 83 paired samples was assessed by qRT-PCR, representative results are shown, and the comparison of the expression levels of CBX8 mRNA between non-metastatic (*n* = 42) and metastatic (*n* = 41) HCCs. **b** CBX8 protein expression for the 83 paired samples was analyzed by western blotting, representative results are shown, and the comparison of the relative expression levels of CBX8 protein between non-metastatic and metastatic HCCs. **c** CBX8 protein expression was analyzed by IHC analysis of 153 paired HCC and normal liver tissues, epresentative results are shown. Semiquantification of CBX8 expression was assessed by IHC of 153 adjacent tissues and primary HCC tissues with or without distant metastasis. **d** The association between CBX8 expression in HCC as determined by IHC and survival time of 153 patients was analyzed by Kaplan–Meier survival analysis. **p < 0.01 compared with adjacent tissues; ^##^*p* < 0.01 compared with the tissues without metastasis. CBX8 Chromobox homolog 8, HCC hepatocellular carcinoma, qRT-PCR quantitative reverse transcription-PCR, mRNA messenger RNA, IHC immunohistochemistry

To determine whether CBX8 expression is correlated with the development and progression of HCC, we first examined CBX8 expression in 83 paired HCC tissues and corresponding adjacent non-tumor tissues by qRT-PCR and western blotting analysis and 153 paired HCC tissues and corresponding adjacent non-tumor tissues by IHC. Compared with the adjacent tissues, the HCC specimens were characterized by overexpressed levels of CBX8 messenger RNA (mRNA) (Fig. 1a and Figure S1A). We also examined the potential correlation of CBX8 expression with HCC disease parameters and tumor morphology. CBX8 overexpression was significantly correlated with distant metastasis in HCC tissues (Fig. 1a). The protein levels of CBX8 in these tissue samples were analyzed by western blotting (Fig. 1b). The protein level of CBX8 was upregulated in HCC samples compared with the normal adjacent tissue samples (Figure S1B). CBX8 protein expression was also significantly correlated with distant metastasis in HCC tissues (Fig. 1b). We then determined the expression levels of CBX8 in Li-7, Sk-Hep-1, SMMC-7721, and the less invasive Hep3B, Huh7, Bel-7402 (Bel), and HepG2 cell lines, as well as normal liver cell line L02. The CBX8 expression was remarkably upregulated in the more invasive HCC cell lines (Figure S1C). These data suggest that the CBX8 upregulation may be relevant to HCC invasiveness and metastasis.

We examined CBX8 protein expression in more HCC samples by IHC. We observed that levels of CBX8 protein were markedly higher in HCC tissues than in normal liver tissues (Fig. 1c). CBX8 protein overexpression consistently and significantly correlated with distant metastasis (Fig. 1c). CBX8 protein expression also significantly correlated with tumor size, microvascular invasion, and differentiation. No statistical connection was found between CBX8 expression and other clinicopathological parameters, such as patient age, gender, and hepatitis B surface antigen (HBsAg) (Table 1). To further assess the relevance of CBX8 expression in HCC, we evaluated the association between CBX8 expression and survival time in HCC by Kaplan–Meier survival analysis (Fig. 1d). The median overall survival time of the high CBX8 expression group was significantly shorter than that of the low CBX8 expression group (*p* < 0.001). These results indicate that CBX8 may have a functional role in the aggressive behavior of HCCs.

**Table 1:** Correlation between CBX8 expression and clinical pathological characteristics of HCC patients

Variables	Total	CBX8 staining		*p* value*
		High	Low	
Gender				
Male	66	36	30	0.417
Female	17	7	10	
Age (years)				
≥50	67	33	34	0.411
<50	16	10	6	
HBsAg				
Positive	60	29	31	0.337
Negative	23	14	9	
Liver cirrhosis				
Yes	55	25	30	0.163
No	28	18	10	
AFP (ng/ml)				
≥20	50	27	23	0.659
<20	33	16	17	
γ-GT (U/L)				
≥50	35	22	13	0.120
<50	48	21	27	
ALT (U/L)				
≥40	42	24	18	0.383
<40	41	19	22	
AST (U/L)				
≥40	38	20	18	1.000
<40	45	23	22	
Tumor diameter (cm)				
≥5	39	29	10	<0.001
<5	44	14	30	
Tumor differentiation				
I + II	51	18	33	<0.001
III	32	25	7	
Tumor stage				
I + II	43	16	27	0.008
III + IV	40	27	13	
TNM stage				
T1 + T2	47	18	29	0.008
T3 + T4	36	25	11	
Metastasis				
Yes	41	26	15	0.049
No	42	17	25	

*CBX8* Chromobox homolog 8, *HCC* hepatocellular carcinoma, *HBsAg* hepatitis B surface antigen, *AFP* α-fetoprotein, *γ-GT* γ-glutamyl transpeptidase, mRNA messenger RNA, *AST* aspartate transaminase, *ALT* alanine aminotransferase,*TNM* tumor node metastasis

*p value is based on the *χ*^2^ test

### CBX8 promotes proliferative capacity of HCC cells both in vitro and in vivo

**Fig. 2:**
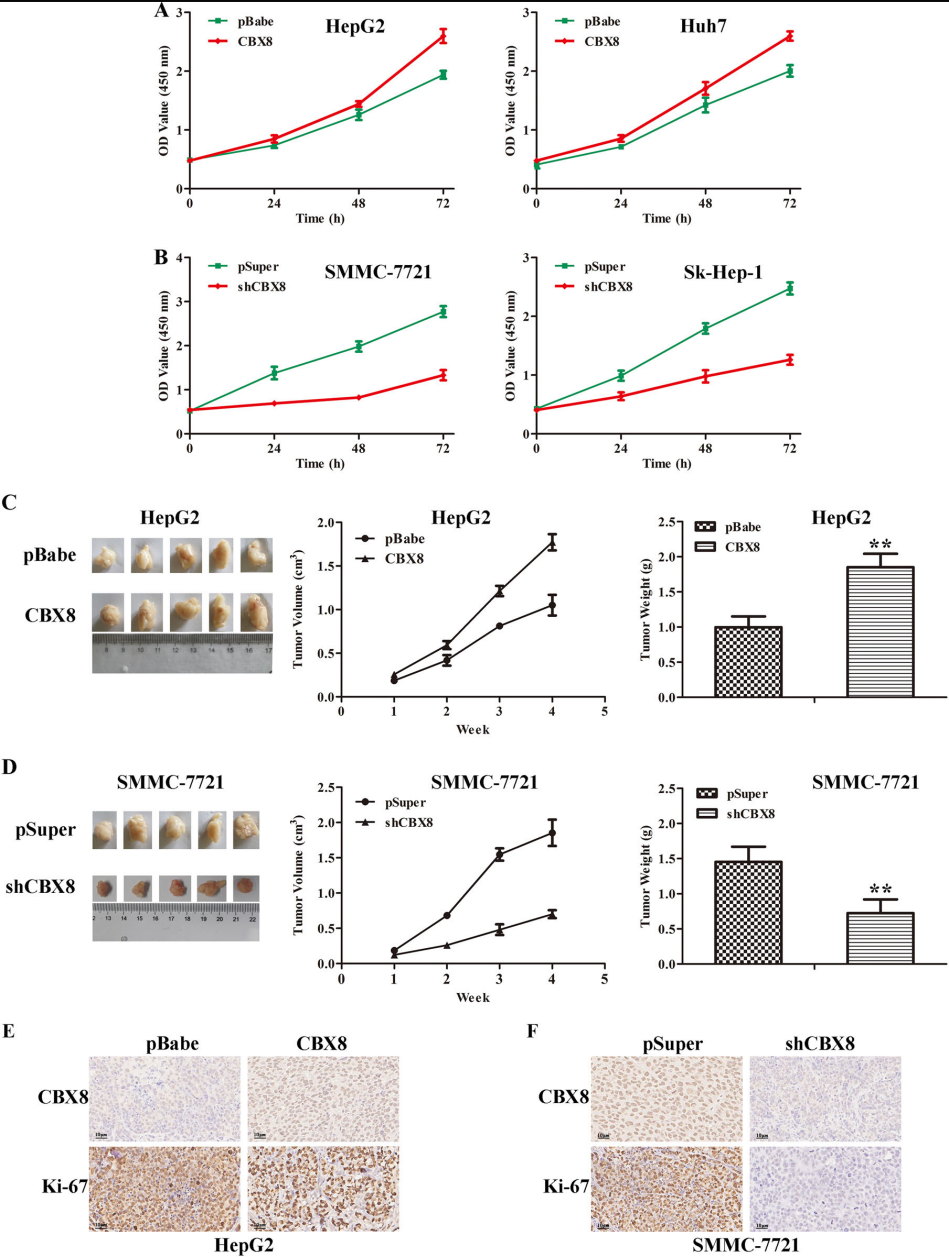
CBX8 promotes proliferative capacity of HCC cells both in vitro and in vivo. **a** Proliferation of HepG2-CBX8, Huh7-CBX8, and their control cells was examined by CCK-8 assay. **b** Proliferation of SMMC-7721-shCBX8, Sk-Hep-1-shCBX8, and their control cells was examined by CCK-8 assay. **c** Images (left), growth (middle), and weight (right) of tumors following subcutaneous injection of HepG2-CBX8 or control cells. **d** Images (left), growth (middle), and weight (right) of tumors following subcutaneous injection of SMMC-7721-shCBX8 or control cells. **e** Representative images of CBX8 and Ki-67 immunostaining in HepG2-CBX8 and the control xenografts. **f** Ki-67 immunostaining in SMMC-7721-shCBX8 and the control xenografts. **p < 0.01. CBX8 Chromobox homolog 8, HCC hepatocellular carcinoma, CCK-8 Cell Counting Kit-8

To explore the potential biological function of CBX8 in HCC tumorigenesis and progression, we retrovirally established stable overexpression of CBX8 in HepG2 and Huh7 cells (designated as HepG2-CBX8 and Huh7-CBX8) (Figure S1D). We analyzed the cellular loss-of-function phenotype via lentivirus-mediated shRNA interference to address the functional importance of CBX8 in HCC development. Three independent shRNAs (shCBX8#1, shCBX8#2, and shCBX8#3) were induced efficient CBX8 knockdown in HCC cells compared with control pSuper- and shScr-infected cells. As shown in Figure S1E, shCBX8#2 was the most effective silencing oligo, and was selected for silencing CBX8 in SMMC-7721 and Sk-Hep-1 cells (designated as SMMC-7721-shCBX8 and Sk-Hep-1-shCBX8) for testing the oncogenic activity of CBX8 in HCC. Expression levels of CBX8 in the transfected cell lines were verified by western blotting (Figure S1F). Compared to controls (HepG2-pBabe and Huh7-pBabe), both HepG2-CBX8 and Huh7-CBX8 cells exhibited significantly increased cell proliferation (Fig. 2a). In contrast, silencing CBX8 in SMMC-7721 and Sk-Hep-1 cells significantly reduced cell proliferation (Fig. 2b). To extend our in vitro observations, we investigated whether CBX8 can regulate the tumorigenic capacity of HCC cells in vivo. HepG2-CBX8, SMMC-7721-shCBX8, and their corresponding control cells were subcutaneously injected into nude mice. Tumor size was measured every week up to 4 weeks. The tumors from HepG2-CBX8 cells grew more rapidly at the implantation site than did tumors derived from the control cells (Fig. 2c). By contrast, silencing CBX8 in the SMMC-7721 cells markedly decreased tumor volume and weight (Fig. 2d). We also detected CBX8 and Ki-67 in these xenografts. In high CBX8 expression xenografts from HepG2-CBX8 and SMMC-7721-pSuper showed relative higher Ki-67 expression than HepG2-pBabe and SMMC-7721-shCBX8 (Fig. 2e, f). Thus, these findings indicate that CBX8 is an important regulator in HCC cells’ proliferation.

### CBX8 regulates the transition between epithelial and mesenchymal phenotypes in HCC cells

**Fig. 3:**
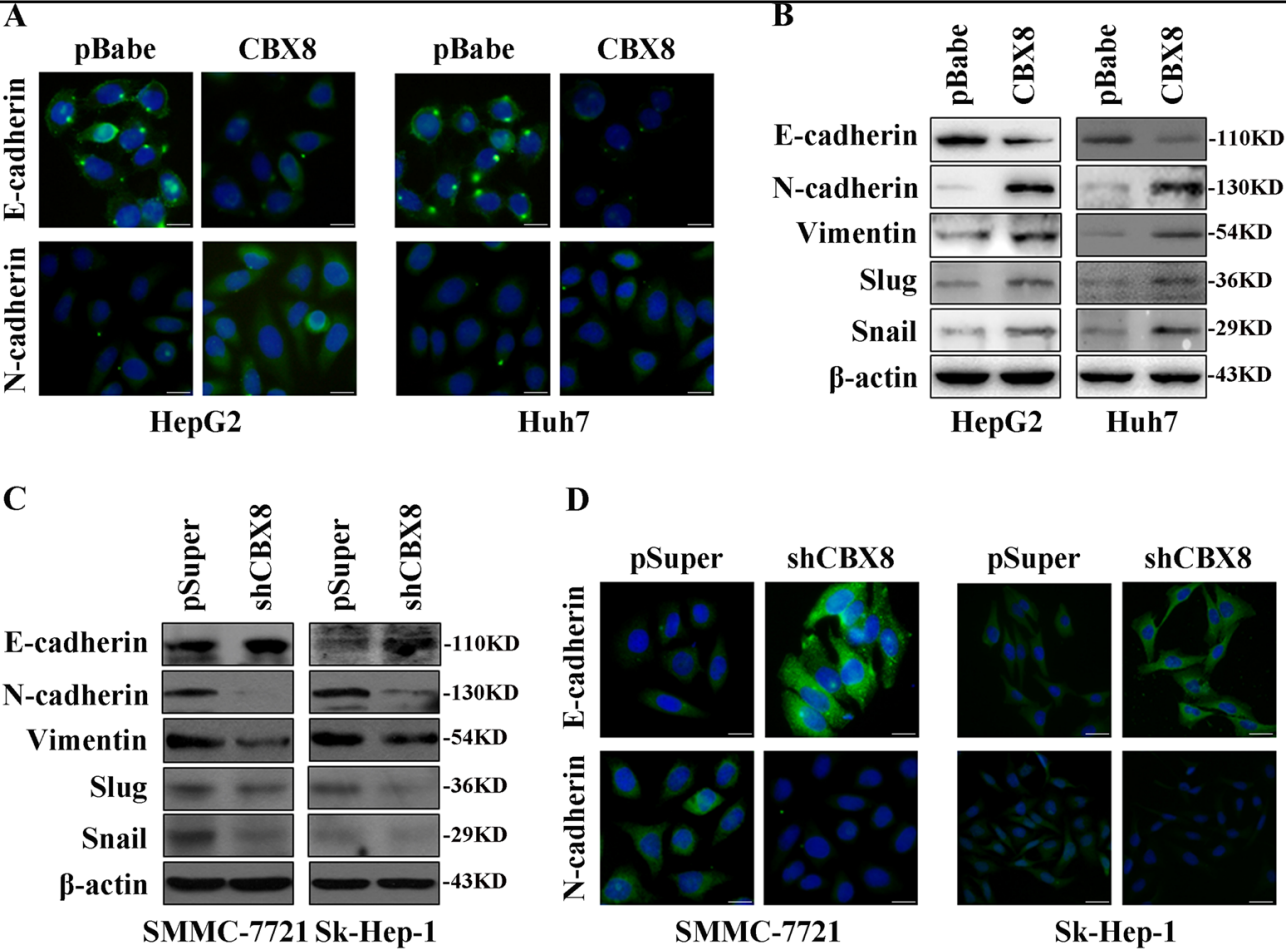
CBX8 regulates the transition between epithelial and mesenchymal phenotypes in HCC cells. **a** Representative phase-contrast images of HepG2 and Huh7 cells showing that CBX8 overexpression modulates the expression of E-cadherin and N-cadherin as analyzed by immunofluorescence staining. **b** Expression of epithelial (E-cadherin) and mesenchymal (N-cadherin, vimentin, Slug, and Snail) markers were analyzed by western blotting in HepG2 and Huh7 cells. **c** Expression of epithelial (E-cadherin) and mesenchymal (N-cadherin, vimentin, Slug, and Snail) markers were analyzed by western blotting in SMMC-7721 and Sk-Hep-1 cells. **d** Representative phase-contrast images of SMMC-7721 and Sk-Hep-1 cells showing that CBX8 shRNA modulates the expression of E-cadherin and N-cadherin as analyzed by immunofluorescence staining. CBX8 Chromobox homolog 8, HCC hepatocellular carcinoma, shRNA short hairpin RNA

We next examined the effect of CBX8 on EMT. We found that overexpression of CBX8 caused a decrease in the level of epithelial marker (E-cadherin), and an increase in the levels of mesenchymal markers (N-cadherin, vimentin, snail, and BMP4) (Fig. 3a, b). Conversely, silencing CBX8 resulted in the upregulation of epithelial markers and downregulation of mesenchymal markers (Fig. 3c, d).

### CBX8 promotes migratory, invasive, and metastatic capacities of HCC cells

**Fig. 4:**
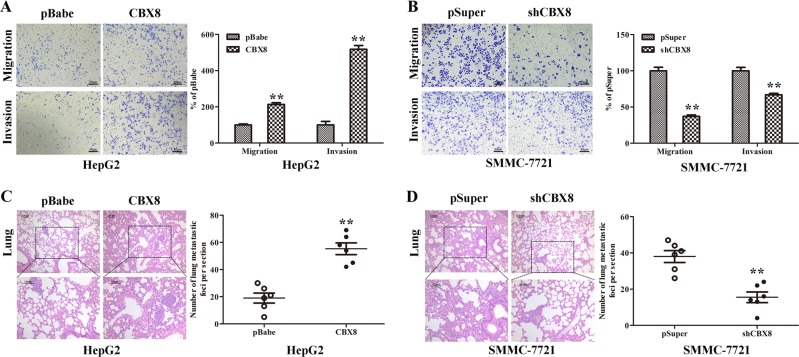
CBX8 promotes migratory, invasive, and metastatic capacities of HCC cells. **a** HepG2-CBX8 and the control cells were subjected to transwell migration and Matrigel invasion assays. Quantification of migrated cells through the membrane and invaded cells through either the membrane or Matrigel for each cell line are shown as a proportion of the vector control. **b** SMMC-7721-shCBX8 and the control cells were subjected to transwell migration and Matrigel invasion assays. Quantification of migrated cells through either the membrane or Matrigel for each cell lines is shown as proportions of their controls. **c** Number of metastatic foci per section in lungs from individual mouse with injection of HepG2-CBX8 or its control cells were determined. A representative stain and quantification of the results are shown. **d** Number of metastatic foci per section in lungs from individual mice injected with SMMC-7721-shCBX8 or its control cells were determined. A representative stain and quantification of the results are shown. ** *p* < 0.01. CBX8 Chromobox homolog 8, HCC hepatocellular carcinoma

The effect of CBX8 on cell migration was first assessed using a Boyden’s chamber assay. Both HepG2-CBX8 and Huh7-CBX8 cells migrated significantly faster through the chamber compared with their counterpart controls; moreover, HepG2-CBX8 and Huh7-CBX8 cells showed a greater degree of invasiveness through Matrigel (Fig. 4a and Figure S2A). However, silencing CBX8 dramatically reduced the migratory and invasive capacity of SMMC-7721 and Sk-Hep-1 (Fig. 4b and Figure S2B). These results indicate that CBX8 promotes migratory and invasive behavior in HCC cells. We then injected HepG2-CBX8, SMMC-7721-shCBX8, and their control cells via the tail vein into nude mice to investigate their metastatic potential by assessing tumor burden in the lung. We found that overexpression of CBX8 not only significantly increased the number of mice with lung tumors, but also increased the number of tumors in the lung (Fig. 4c and Table S1). Silencing CBX8 in SMMC-7721 cells inhibited this behavior, both in terms of the number of mice with lung tumors and the number of tumors in the lungs (Fig. 4d and Table S1). Therefore, these in vivo data further support the critical pro-tumorigenic role for CBX8 in HCC.

### CBX8 promotes the emergence of stem cell-like behavior in HCC cell lines

**Fig. 5:**
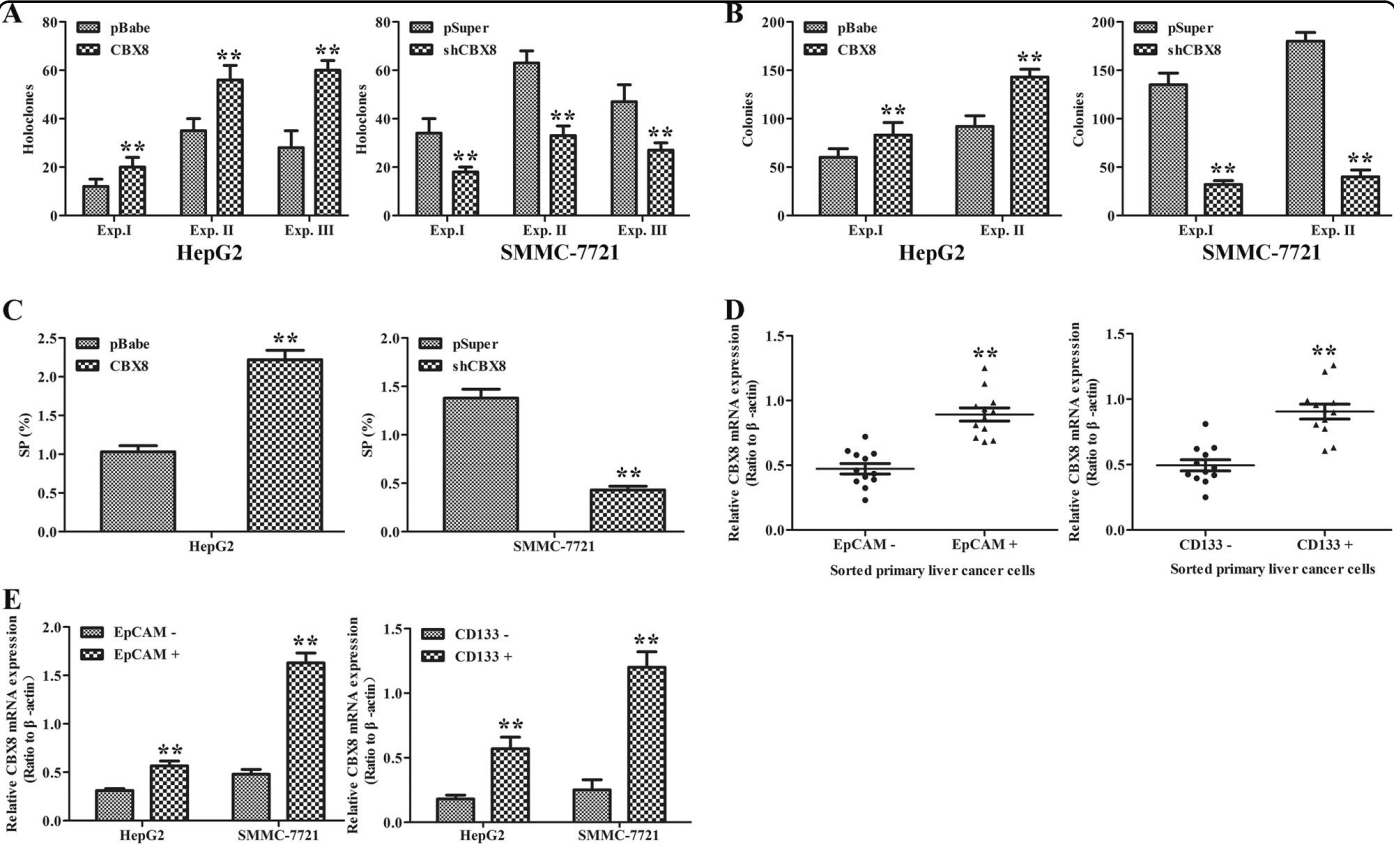
CBX8 promotes stem cell-like behavior of HCC cells. **a** Holoclone assays in HepG2 cells transfected with pBabe or pBabe-CBX8, and SMMC-7721 cells transfected with pSuper or pSuper-shCBX8 were used in three experiments (Exp. I, 100 cells/well scored on d 9; Exp. II, 100 cells/well scored on d 13; Exp. III, 500 cells/well scored on d 7). **b** Clonogenic assay (Exp. I, 1250 cells/well scored on d 5; Exp. II, 25,000 cells/well scored on d 5) in HepG2 cells transfected with pBabe or pBabe-CBX8 and SMMC-7721 cells transfected with pSuper or pSuper-shCBX8. Two experiments were performed (Exp. I, 1250 cells/well scored on d 5; Exp. II, 25,000 cells/well scored on d 5). **c** SP population in CBX8 overexpression and silenced HCC cells was determined by Hoechst 33342 efflux assay. **d** The expression of CBX8 mRNA in sorted primary EPCAM− and EPCAM+ liver cancer cells was determined by qRT-PCR (left). The expression of CBX8 mRNA of sorted primary CD133− and CD133+ liver cancer cells was determined by qRT-PCR (right). **e** Levels of CBX8 mRNA in magnetically sorted EPCAM+ and EPCAM− were measured by qRT-PCR in HepG2 and SMMC-7721 cells (left). Levels of CBX8 mRNA in magnetically sorted CD133+ and CD133− were measured by qRT-PCR in HepG2 and SMMC-7721 cells (right). **p < 0.01. CBX8 Chromobox homolog 8, HCC hepatocellular carcinoma, qRT-PCR quantitative reverse transcription-PCR, mRNA messenger RNA

Increasing evidence has linked EMT with the acquisition of molecular and functional traits of stem cells in normal and neoplastic cell populations. Thus, we performed holoclone, colonogenic, and sphere-forming assays to determine whether CBX8 regulates certain stem cell-associated properties. We first established stringent assay conditions in which clones, colonies, and spheres were all clonal origin. CBX8 overexpression promoted holoclone formation, clonogenic capacity, and sphere establishment in HepG2 (Fig. 5a–c) and Huh7 (Figure S3A–C) cells. By contrast, silencing CBX8 in both SMMC-7721 (Fig. 5a–c) and Sk-Hep-1 (Figure S3D–F) inhibited these capacities. Liver stem-like cells within tumors (i.e., tumor-initiating cells (T-ICs)) are characterized by several cell marker molecules, such as EpCAM and CD133. We further examined the expression of CBX8 in sorted primary EpCAM+ or CD133+ liver cancer cells. CBX8 showed significantly elevated expression in sorted primary EpCAM+ or CD133+ liver cancer cells relative to matched EpCAM− or CD133− cells (Fig. 5d). Similar results were also obtained in magnetically sorted EpCAM+ or CD133+ HepG2 and SMMC-7721 cells (Fig. 5e). Meanwhile, ectopic expression of CBX8 facilitated EpCAM and CD133 expression in HepG2 and Huh7 (Figure S3G), whereas CBX8 knockdown suppressed both EpCAM and CD133 in SMMC-7721 and Sk-Hep-1 (Figure S3H). Thus, these results indicate that CBX8 can promote properties associated with stemness.

### CBX8 regulates BMP4 expression through H3K27 trimethylation

**Fig. 6:**
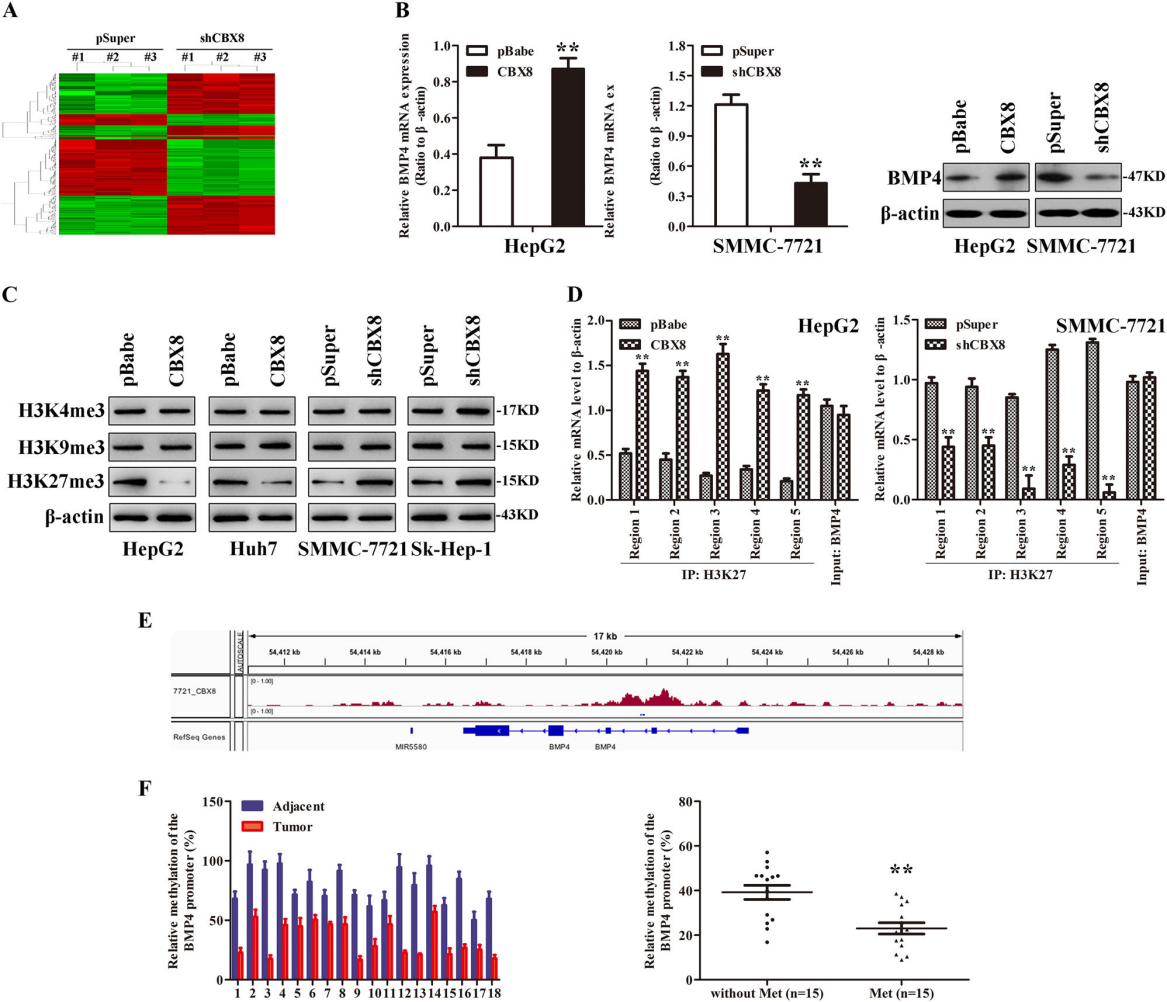
CBX8 regulates BMP4 expression through H3K27 trimethylation. **a** Supervised hierarchical clustering of the differentially expressed genes after CBX8 silencing in SMMC-7721 cells. **b** BMP4 mRNA expression was measured by qRT-PCR and BMP4 protein expression was measured by western blotting in HCC cells with CBX8 overexpression or knockdown. **c** H3K4me3, H3K9me3, and H3k27me3 was measured by western blotting. **d** Presentation of five regions relative to the BMP4 transcriptional start site used as primers to test histone occupied abundance. **e** BMP4 is one of the genes that bind on CBX8 protein by ChIP-seq. **f** The relative methylation of the BMP4 promoter in HCC tissues. Representative results are shown between adjacent liver tissues and HCC tissues, and the comparison of the relative methylation of the BMP4 promoter between HCC tissues with or without metastasis. **p < 0.01. CBX8 Chromobox homolog 8, BMP4 bone morphogenetic protein 4, mRNA messenger RNA, qRT-PCR quantitative reverse transcription-PCR, ChIP-seq chromatin immunoprecipitation-sequencing

We performed gene expression profiling on SMMC-7721-shCBX8 and its control cells to better understand the mechanisms by which CBX8 engaged in the EMT program and consequent T-IC-like functionality. Microarray analyses identified several genes significantly and differentially expressed after CBX8 knockdown, including downregulation of BMP4 (Fig. 6a). Furthermore, gene set enrichment analysis indicated that neoplasm metastasis and invasion, cell movement, mammary stem cell, and BMP4-related gene signatures were significantly enriched in CBX8-overexpressing cells (data not shown). These results confirm that CBX8 regulates EMT, stemness, and cancer invasion and metastasis, which may be mediated by BMP4. HepG2-CBX8 cells exhibited markedly upregulated expression of BMP4 at both the mRNA and protein level, whereas silencing CBX8 in SMMC-7721 cells dramatically decreased BMP4 expression at both the mRNA and protein level (Fig. 6b). These findings imply that CBX8 may regulate BMP4 expression at the transcriptional level. Histone modification patterns were measured after modulation of CBX8 to determine whether CBX8 activity was associated with specific histone modifications in HCC cells. Among histones H3K4me3, H3K9me3, and H3K27me3, we found that only H3K27me3 was affected by changes in CBX8 expression. Ectopic expression of CBX8 decreased H3K27me3, while silencing of CBX8 increased this modification (Fig. 6c).

We next determined whether CBX8 expression was correlated with H3K27me3 modification at the BMP4 gene promoter in HCC cells. ChIP assays were performed in SMMC-7721-shCBX8 and control cells. Antibodies against HeK27me3 were used to pull down the chromatin complex, and five pairs of primers against the BMP4 gene promoter region were used to assess the occupancy of the BMP4 gene promoter. CBX8 expression was associated with decreased H3K27m3 levels at these five regions of the BMP4 gene promoter in HepG2 and SMMC-7721 cells (Fig. 6d). We used CBX8 to perform ChIP-sequencing (ChIP-seq), and found that CBX8 was not present at the BMP4 promoter site (Fig. 6e).

The methylation of CpG island in the area of the tumor oncogene promoter is one of the most important factors responsible for cancer^17^. BMP4 is an oncogene with a length of 8535 bp and is located on the 14q22.2 chromosome. To investigate whether BMP4 expression is regulated by DNA methylation, we determined the degree of methylation of the 5′-flanking sequence of the BMP4 gene. Using CpG island analysis software Methyl Primer Express Version 1.0 (http://www.appliedbiosystems.com), we found a CpG island of 157 bp in length, and a total of 13 CpG sites were analyzed (Figure S4A). Methylation analysis indicated that the level of methylated CpG sites of BMP4 in HepG2 and Huh7 were higher than that in SMMC-7721 and Sk-Hep-1 (Figure S4B), suggesting that the methylation level of the BMP4 promoter correlates with HCC metastasis. We also analyzed the methylation level of BMP4 promoter in 30 HCC patients, and found that the methylation level of the BMP4 promoter in adjacent tissue is much higher than in tumor tissue, and lower methylation level in HCC tissue was associated metastasis (Fig. 6f). These results indicate that CBX8 induces transcriptional activation of BMP4 through decreasing H3K27me3 in the BMP4 gene promoter in HCC cells.

### BMP4 is a mediator for CBX8-induced EMT, migration, invasion, stemness, and in vivo metastastatic capacity in HCC cells via Smads and MAPK pathway

**Fig. 7:**
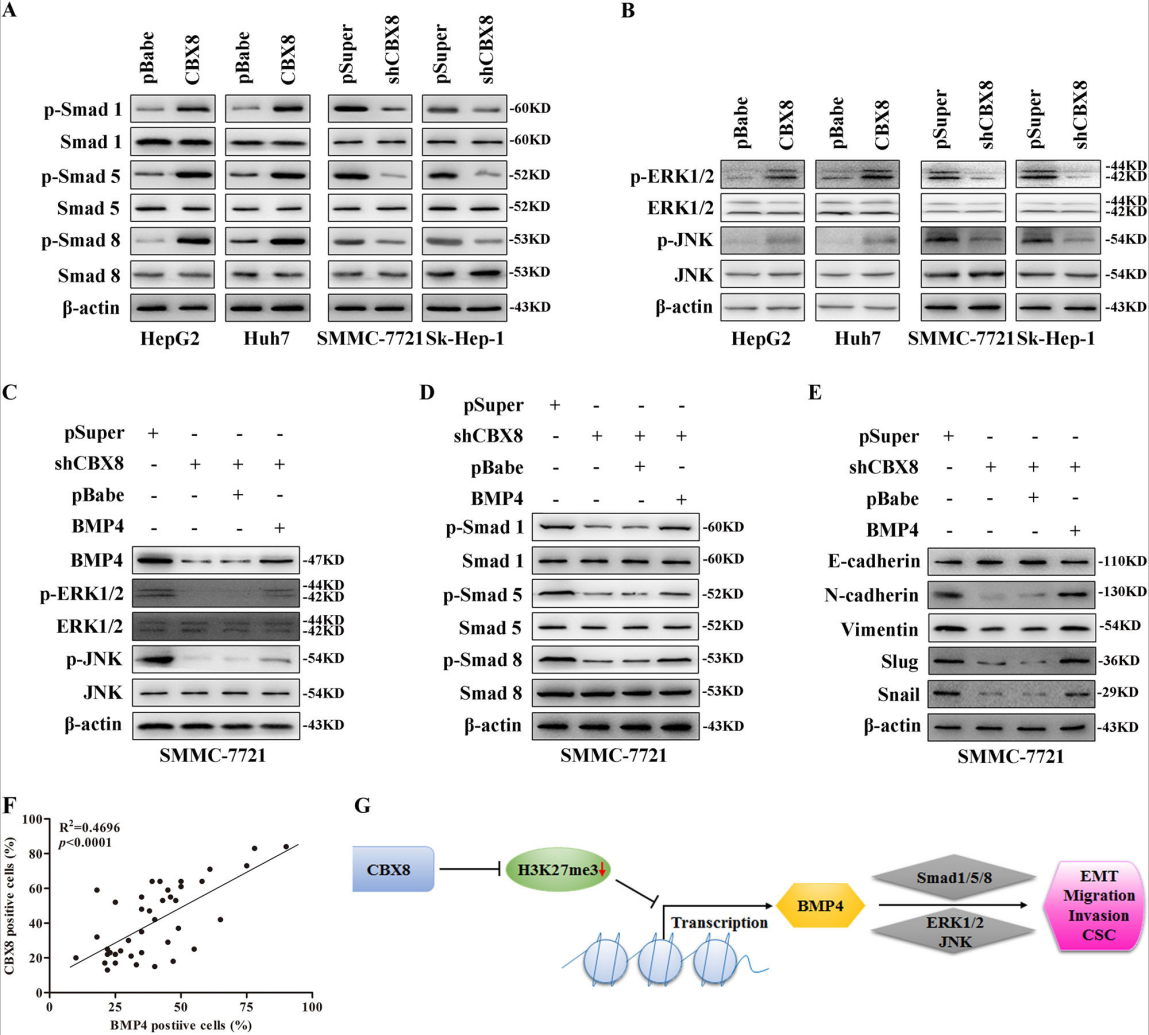
BMP4 is a mediator of CBX8-induced EMT, migration, invasion, stemness, and in vivo metastasis capacity via Smads and MAPKs in HCC cells. **a** Expression of (p-) Smad1/5/8 were measured by western blotting. **b** Expression of (p-) ERK and (p-) JNK were measured by western blotting. **c**–**e** Expression of BMP4, (p-) ERK, (p-) JNK, (p-) Smad1/5/8, E-cadherin, N-cadherin, Vimentin, Slug, and Snail were measured by western blotting. **f** BMP4 expression was positively correlated with CBX8 expression in HCC tissues. **g** Schematic illustration of the proposed function of CBX8 in HCC metastasis and stemness. CBX8 Chromobox homolog 8, BMP4 bone morphogenetic protein 4, EMT epithelial–mesenchymal transition, MAPK mitogen-activated protein kinase, p- phosphorylated, ERK extracellular signal-regulated kinase, JNK c-JUN N-terminal kinase, HCC hepatocellular carcinoma

To better understand the mechanisms by which CBX8 engaged in HCC progression, we further analyzed CBX8-knockdown gene expression profiling of SMMC-7721-shCBX8 and corresponding control cells. Gene set enrichment analysis showed that CBX8 knockdown modified expression of a cluster of genes associated with cell proliferation, movement and metastasis, and apoptosis, including tumor growth factor-β and MAPK signaling pathway genes (Figure S4C). Western blot analysis showed that CBX8 expression upregulated phosphorylated (p)-Smad1/5/8, and increased p-ERK1/2 and p-JNK HepG2 and Huh7 cells. Conversely, CBX8 silencing downregulated p-Smad1/5/8, and decreased p-ERK1/2 and p-JNK in SMMC-7721 and Sk-Hep-1 cells (Fig. 7a, b).

To test whether CBX8 induced HCC progression is mediated by BMP4, we overexpressed BMP4 in SMMC-7721-pSuper and SMMC-7721-shCBX8 cells. BMP4 reactivated ERK1/2 and JNK, and increased p-Smad1/5/8 (Fig. 7c, d). Moreover, extrinsic BMP4 gene expression in SMMC-7721-shCBX8 cells reversed EMT progression induced by silencing CBX8 (Fig. 7e). These phenomena were accompanied by a promotion of migratory and invasive capacities (Figure S5A). SMMC-7721-shCBX8 cells, with or without overexpression of BMP4, were injected into nude mice via the tail vein to verify whether BMP4 mediates CBX8-induced metastasis in vivo. We observed that BMP4 significantly increased the number of mice with distant metastasis (Table S2). These in vivo results demonstrate a critical role for BMP4 in mediating CBX8-promoted metastatic behavior in HCC cells. Moreover, BMP4 increases holoclone formation, clonogenic capacity, and percentage of SP cells (Figure S5B–D) of SMMC-7721-shCBX8 cells. Furthermore, silencing BMP4 inhibited SMMC-7721 cells' proliferation, colony formation, and EMT, while overexpression of CBX8 did not rescue these functions caused by BMP4 knockdown (Figure S6A–C).

We also analyzed BMP4 expression in the same human HCC tissue to identify any clinical correlation of CBX8 and BMP4. There was a high positive correlation between CBX8 and BMP4 expression (Fig. 7f). These results are consistent with the above in vitro and in vivo data and provide further support that BMP4 mediates CBX8-induced EMT, migration, invasion, and stemness via Smads and MAPK pathway in HCC cells (Fig. 7g).

## Discussion

Many effects have been done to reveal the significance of CBX8 in cancer development and progression. While it still remains, lots of function and mechanism need to be studied. By using a variety of in vivo and in vitro approaches, as well as samples from 153 patients with HCC, we characterized the clinical significance of CBX8 in HCC and the mechanistic role of CBX8 in regulating HCC cell metastasis. CBX8 overexpression in HCC cells induces EMT, migration, invasion, and stem cell-like traits in vitro and enhances the stem cell-like and metastatic capacity in vivo. By contrast, silencing of CBX8 reverses these events in otherwise aggressive and invasive HCC cells. We also demonstrated that a mechanistic link exists between CBX8 and BMP4 through CBX8-mediated regulation of H3K27 trimethylation, which subsequently leads to transcriptional upregulation of BMP4 expression. Overexpression of BMP4 reverses the inhibition induced by CBX8 silencing of the aggressive and invasive properties of HCC cells, which indicates a direct role for BMP4 in this process. Thus, we propose a model for CBX8 activation of EMT, stemness, and metastasis in HCC cells, at least partly through transcriptional regulation of BMP4 (Fig. 7f).

The role of CBX8 as an oncogene in cancer development is supported by the high expression of CBX8 in leukemia, colon, and breast carcinomas relative to normal tissues^7,18,19^. We determined that CBX8 is also expressed at high levels in HCC and that HCC cells expressing high levels of CBX8 display an EMT phenotype, which involves the associated stimulatory effects on in vitro migration and invasion. These results are consistent with what Zhang et al.^20^ found recently. Our study describes a novel function of CBX8 in HCC metastasis of promoting two essential characteristics of metastatic disease in HCCs: EMT and stemness. EMT and stem cell-like properties are essential for HCC cells to disseminate from adjacent tissues and seed new tumors in distant sites^21,22^. Our results demonstrated that CBX8 regulates these two essential characteristics of metastatic disease and that CBX8-induced processes are reversible with the suppression of CBX8 expression. CBX8 also significantly enhances the self-renewal and tumor-initiating capability of HCC cells, as well as properties that are essential to T-ICs. Thus, CBX8 promotes characteristics of migrating T-ICs, which have been proposed as a model for cancer progression and metastasis^23^. We demonstrated that these characteristics, which are induced by CBX8 in vitro, culminate in increased numbers of distant metastases in vivo. These empirical findings provide a mechanistic framework to explain clinical observations that HCC patients with high levels of CBX8 in tissue samples have a higher probability of exhibiting distant metastases, as well as significantly shorter overall survival rates.

BMP family members comprise multifunctional cytokines that play critical roles during embryonic development, organogenesis, and adult tissue remodeling by regulating cell proliferation, growth, differentiation, and apoptosis^24^. We identified BMP4 as an effective mediator of CBX8-induced metastasis and stem cell-like traits in HCC cells. To our knowledge, this is the first demonstration of a mechanistic connection between CBX8 and BMP4. We also determined that modulation of CBX8 expression alters the methylation status of H3K27 at the BMP4 gene promoter, which in turn transcriptionally controls the expression of BMP4. The methylation of the histone lysines H3K4, H3K9, H3K27, H3K36, H3K79, and H4K20 plays an essential role in the regulation of key biological processes, such as cell cycle progression, transcription, and DNA repair^25–30^. In human cells, H3K27me3, catalyzed by methyltransferase, is enriched at silent gene promoters in mammalian cells, as well as in female mammals who have inactivated X chromosomes, and plays an important role in modulating the expression of developmentally regulated genes^31^. Thus, we conclude that CBX8 may transcriptionally activate BMP4 expression by decreasing H3K27 trimethylation at the BMP4 gene promoter, which consequently promotes migration and stemness in vitro and metastasis in vivo.

BMP4 signaling involves its binding to Type I and Type II serine/threonine kinase receptors and the subsequent activation of Smad-dependent and Smad-independent pathways, resulting in the regulation of a plethora of genes related to cell function^32^. The Smad pathway is the canonical pathway thought to mediate the anti-proliferative or anti-metastasis effects of various BMP ligands. Accumulating evidence indicates that the tumorigenic effects of BMP4 are transmitted through Smad-independent pathways, including MAPK signaling pathways^33,34^. Interestingly, as the RNA-seq analysis data shown in the work by Zhang et al.,^20^ MAPK was also found significantly activated by CBX8. We determined that CBX8 activates the phosphorylation of Smad1/5/8, ERK1/2, and JNK, and that BMP4 mediates this activation. Thus, targeting of the enzymatic activity of CBX8 may provide a novel approach to therapy against HCC by modulating a variety of downstream pathways.

Our results establish CBX8 as a critical driver of HCC stem cell-like and metastatic behaviors and characterize its role in modulating BMP4 expression. These findings have implications for the targeting of CBX8 as an approach to HCC prognosis and treatment.

## Supplementary information

Figure S1

Figure S2

Table S1

Figure S3

Figure S4

Figure S5

Table S2

Figure S6

Supplementary figure legends
