# Supplementary figures and images for: CBX8 exhibits oncogenic properties and serves as a prognostic factor in hepatocellular carcinoma
Source: Cell Death Dis. 2019 Jan 18;10(2):52. doi: 10.1038/s41419-018-1288-0 (PMC6361915; doi:10.1038/s41419-018-1288-0)

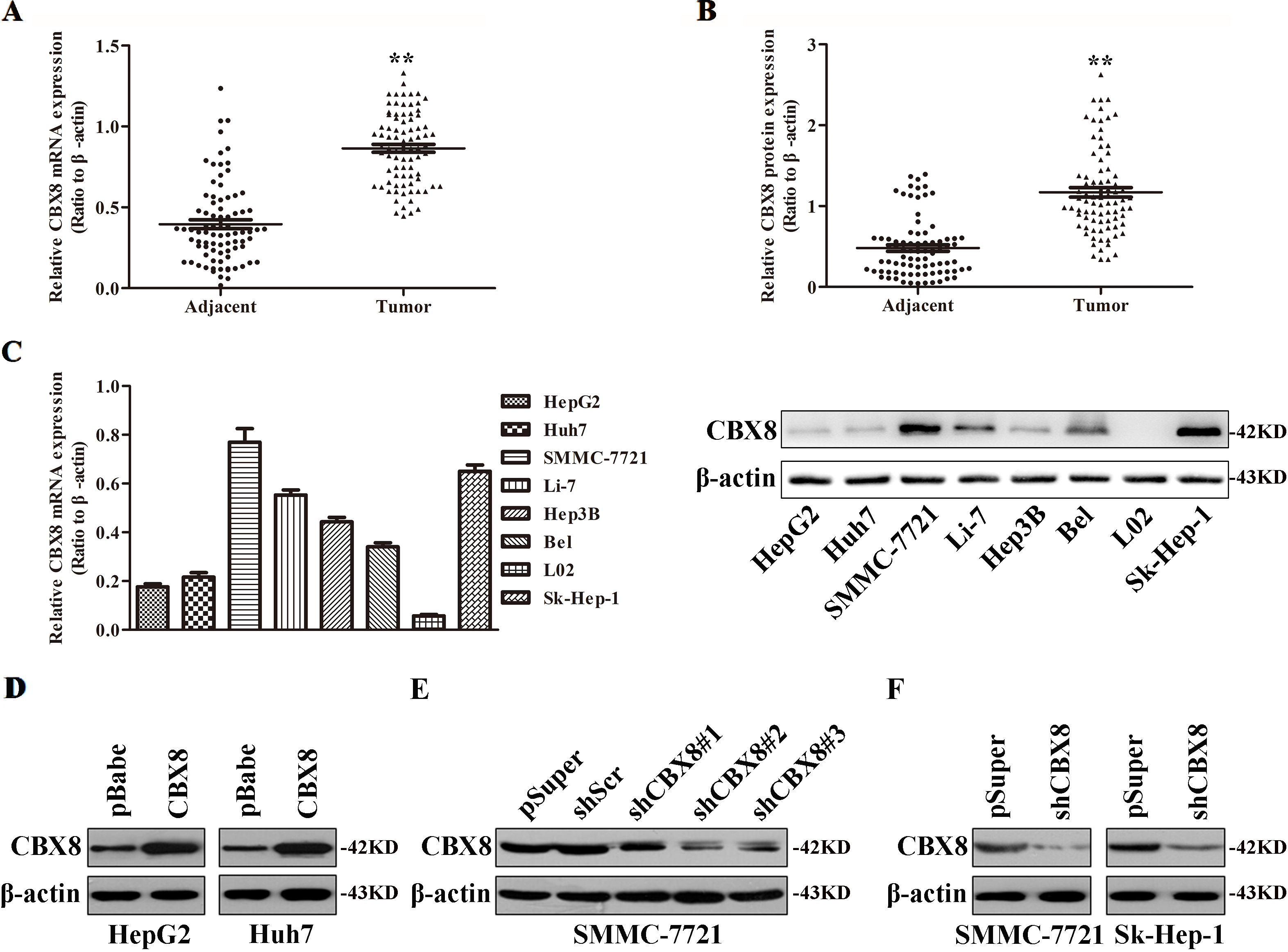

Supplement: Supplementary file 1 — Figure S1 [file 41419_2018_1288_MOESM1_ESM.jpg]

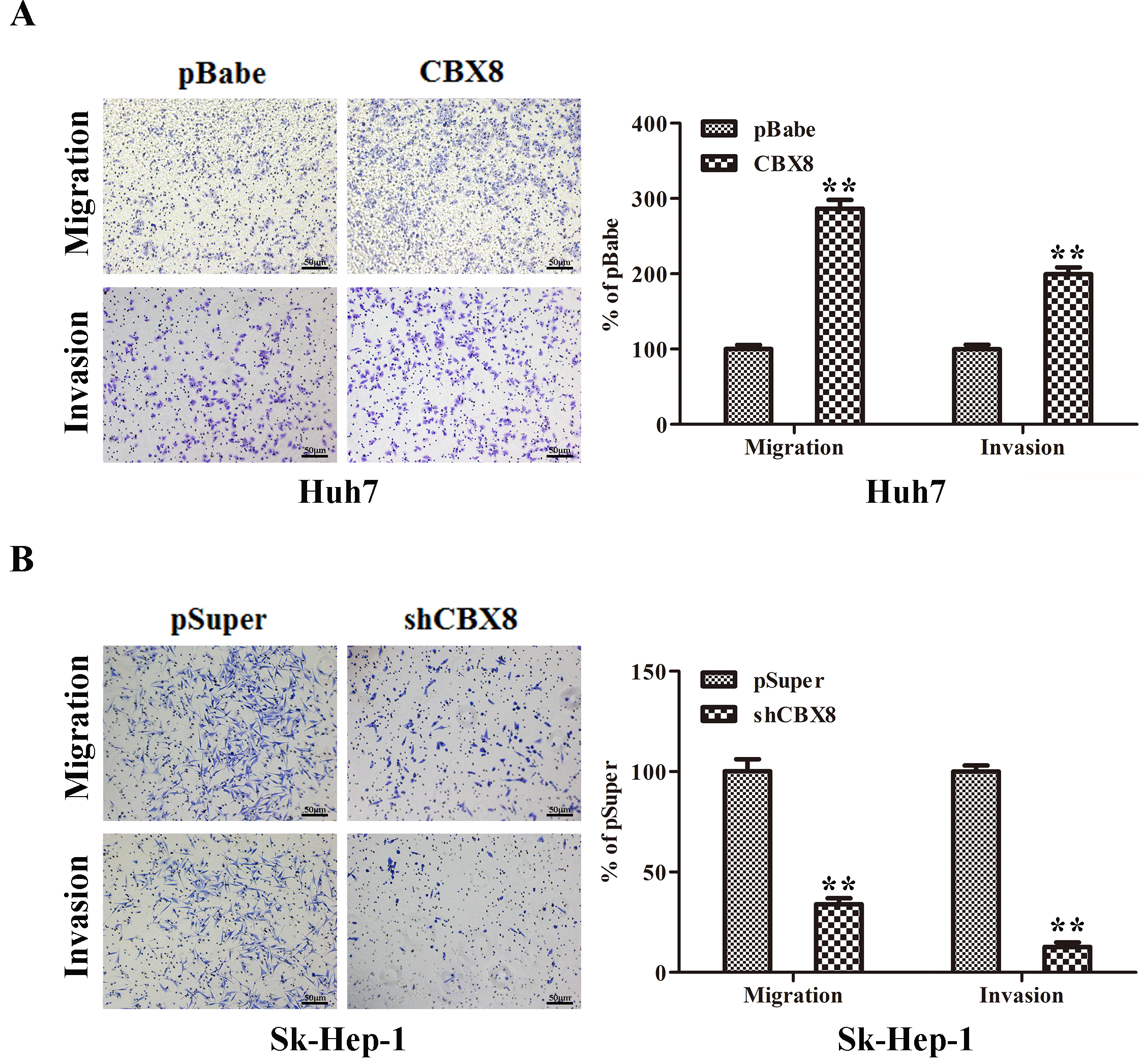

Supplement: Supplementary file 2 — Figure S2 [file 41419_2018_1288_MOESM2_ESM.jpg]

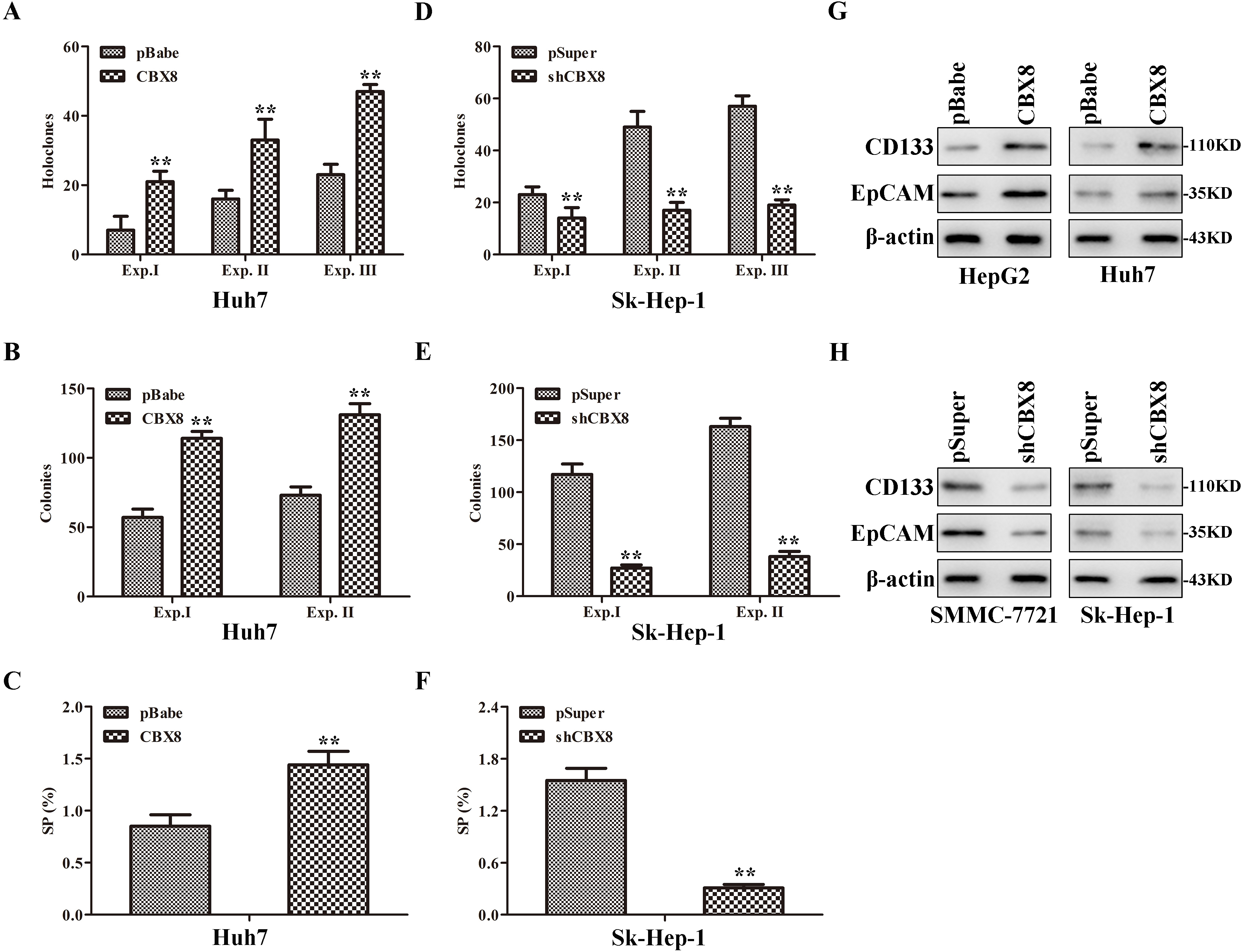

Supplement: Supplementary file 4 — Figure S3 [file 41419_2018_1288_MOESM4_ESM.jpg]

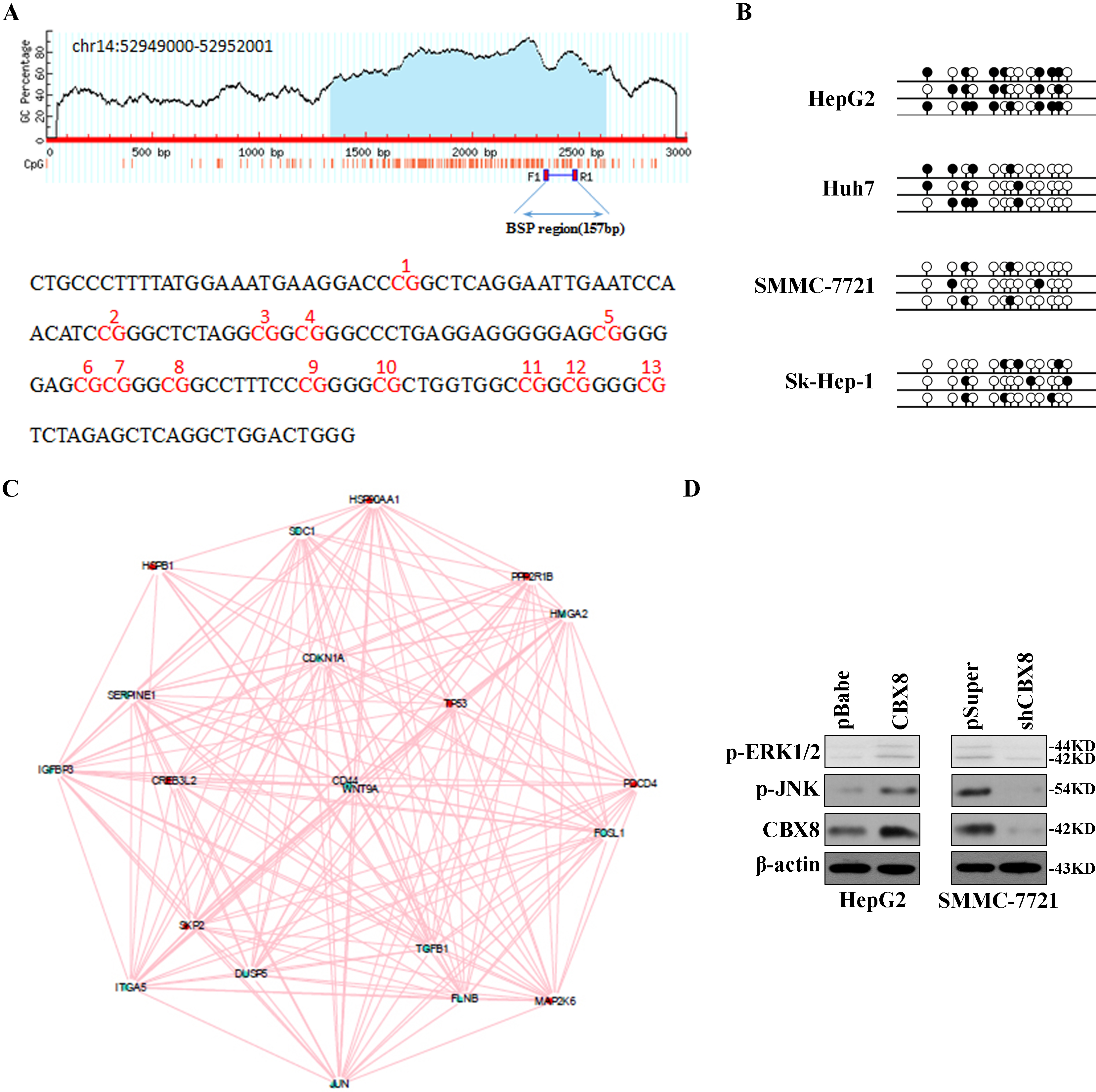

Supplement: Supplementary file 5 — Figure S4 [file 41419_2018_1288_MOESM5_ESM.jpg]

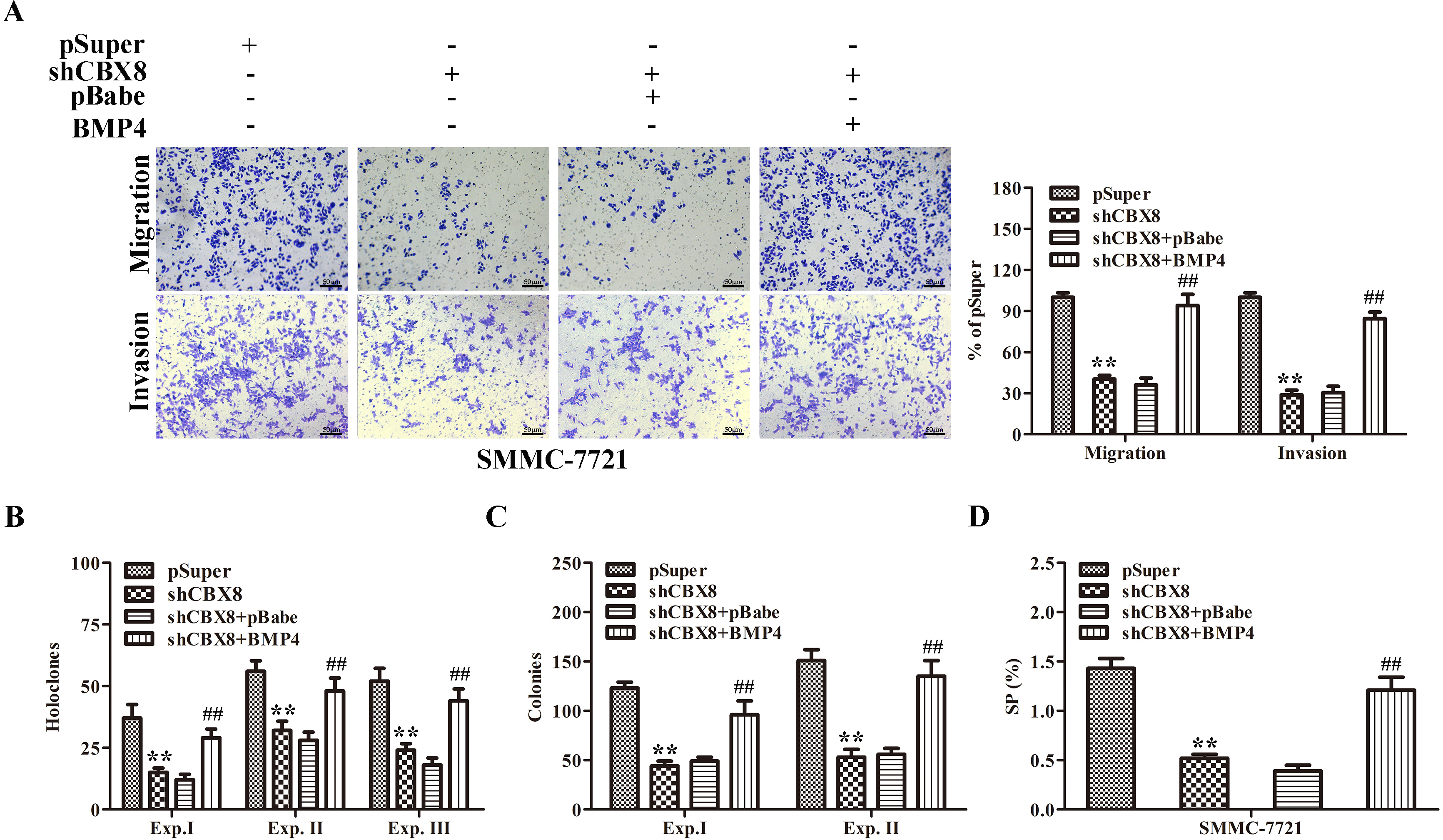

Supplement: Supplementary file 6 — Figure S5 [file 41419_2018_1288_MOESM6_ESM.jpg]

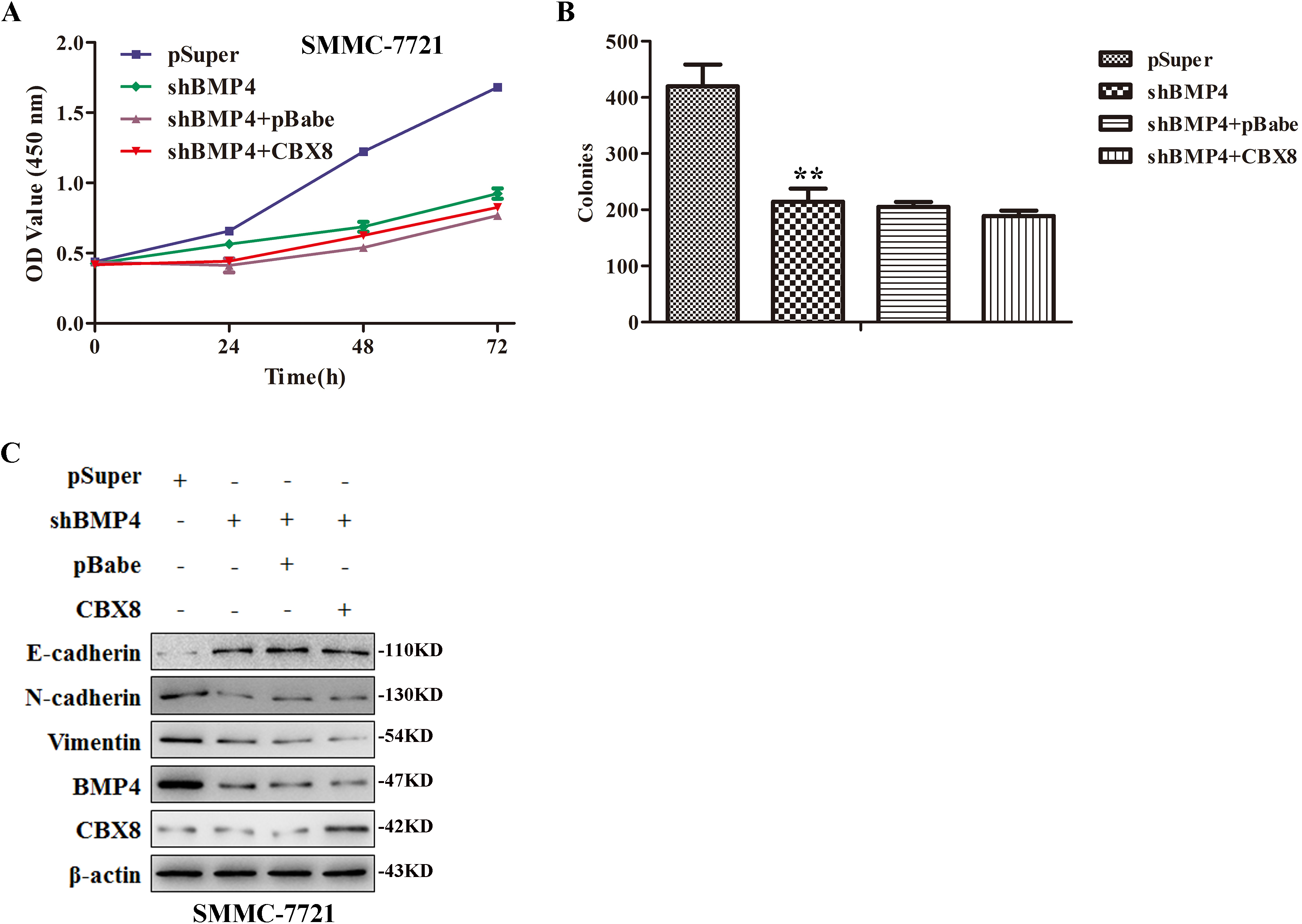

Supplement: Supplementary file 8 — Figure S6 [file 41419_2018_1288_MOESM8_ESM.jpg]
