# Supplementary material for: CBX8 exhibits oncogenic properties and serves as a prognostic factor in hepatocellular carcinoma
Source: Cell Death Dis. 2019 Jan 18;10(2):52. doi: 10.1038/s41419-018-1288-0 (PMC6361915; doi:10.1038/s41419-018-1288-0)
Supplement: Supplementary file 3 — Table S1 [file 41419_2018_1288_MOESM3_ESM.docx]

**Number of mice with distant metastasis injected with different transfected HepG2 and SMMC-7721 cells**

|  | **Number of mice with distant metastasis** |
| --- | --- |
| HepG2-pBabe | 1/6 |
| HepG2-CBX8 | 4/6 |
| SMMC-7721-pSuper | 5/6 |
| SMMC-7721-shCBX8 | 2/6 |
