# Supplementary material for: CBX8 exhibits oncogenic properties and serves as a prognostic factor in hepatocellular carcinoma
Source: Cell Death Dis. 2019 Jan 18;10(2):52. doi: 10.1038/s41419-018-1288-0 (PMC6361915; doi:10.1038/s41419-018-1288-0)
Supplement: Supplementary file 7 — Table S2 [file 41419_2018_1288_MOESM7_ESM.docx]

**Number of mice with distant metastasis injected with different transfected SMMC-7721 cells**

| **SMMC-7721-shCBX8** | **Number of mice with distant metastasis** |
| --- | --- |
| pBabe | 2/6 |
| BMP4 | 5/6 |
