# Supplementary material for: CBX8 exhibits oncogenic properties and serves as a prognostic factor in hepatocellular carcinoma
Source: Cell Death Dis. 2019 Jan 18;10(2):52. doi: 10.1038/s41419-018-1288-0 (PMC6361915; doi:10.1038/s41419-018-1288-0)
Supplement: Supplementary file 9 — Supplementary figure legends [file 41419_2018_1288_MOESM9_ESM.docx]

**Figure S1. CBX8 is highly expressed in HCC tissues and cancer cell lines.**

A: Comparison of the relative mRNA levels of CBX8 between 83 adjacent liver tissues and HCC tissues. B: Comparison of the relative protein levels of CBX8 between 83 adjacent liver tissues and HCC tissues. C: The expression of CBX8 mRNA was measured by RT-PCR in HepG2, Huh7, SMMC-7721, Li-7, Hep3B, Bel and Sk-Hep-1, as well as normal liver cells L02. Relative CBX8 protein expression in these cell lines was assessed by western blotting. D: Expression of CBX8 was measured by western blotting in HepG2-CBX8, Huh7-CBX8 and their control cells. E: Expression of CBX8 was measured by western blotting of three different SMMC-7721 CBX8 knockdown (SMMC-7721-shCBX8#1, #2 and #3) and the control cells. F: CBX8 knockdown SMMC-7721 and Sk-Hep-1 cells were prepared using shCBX#2 (SMMC-7721-shCBX8 and Sk-Hep-1-shCBX8), expression of CBX8 was measured by western blotting. **, P<0.01.

**Figure S2. CBX8 promotes migratory and invasive capacities of Huh7 and Sk-Hep-1 cells.**

A: Huh7-CBX8 and the control cells were subjected to transwell migration and matrigel invasion assays. Representative wells (left) and quantification of migrated and invaded cells (right panel) are shown. B: Sk-Hep-1-shCBX8 and the control cells were subjected to transwell migration and matrigel invasion assays. Representative wells and quantification of migrated and invaded cells are shown. **, p<0.01.

**Figure S3. CBX8 promotes the emergence of stem cell-like behavior in Huh7 and Sk-Hep-1 cells.**

A: Holoclone assays of Huh7 cells. Cells transfected with pBabe or pBabe-CBX8 were used in three experiments (Exp. I, 100 cells/well scored on d 9; Exp. II, 100 cells/well scored on d 13; Exp. III, 500 cells/well scored on d 7). B: Clonogenic assays of Huh7 cells. Two experiments were performed (Exp. I, 1,250 cells/well scored on d 5; Exp. II, 25,000 cells/well scored on d 5). C: SP population of Huh7-CBX8 and its control cells was determined by Hoechst 33342 efflux assay. D: Holoclone assays of Sk-Hep-1 cells. Cells transfected with pSuper or pSuper-shCBX8 were used in three experiments (Exp. I, 100 cells/well scored on d 9; Exp. II, 100 cells/well scored on d 13; Exp. III, 500 cells/well scored on d 7). E: Clonogenic assay of Sk-Hep-1 cells. Two experiments were performed (Exp. I, 1,250 cells/well scored on d 5; Exp. II, 25,000 cells/well scored on d 5). F: SP populations of Sk-Hep-1-shCBX8 and its control cells were determined by Hoechst 33342 efflux assay. G. EpCAM and CD133 expression levels were measured by western blotting in HepG2 and Huh7 cells. H. EpCAM and CD133 expression levels were measured by western blotting in SMMC-7721 and Sk-Hep-1 cells **, p<0.01.

**Figure S4. CBX8 regulates BMP4 and MAPK signaling pathway.**

A: Analysis result of CpG islands in ﬂanking sequence of goat BMP4 gene which was performed by the CpG island analysis software Methyl Primer Express Version 1.0 (http://www.appliedbiosystems.com). Distribution of CpG sites is shown below and marked in red. The nucleotide positions are marked based on the BMP4 sequence of human in GenBank. BSP, bisulﬁte sequencing PCR. B: Detection of the methylation sites of BMP4 promoter in HepG2, Huh7, SMMC-7721 and Sk-Hep-1 cells. C: Gene set enrichment analysis was carried out using ConceptGen. D. Expression of p-ERK1/2 and p-JNK was detected by western blotting in xenograft tissues.

**Figure S5. BMP4 is a mediator for CBX8-induced migration, invasion and in vivo metastasis capacity in HCC cells.**

A: Migratory and invasive capacities were measured by transwell migration and matrigel invasion assays in SMMC-7721 cells. B: Holoclone asssay was performed in SMMC-7721 cells. Cells were used in three experiments (Exp. I, 100 cells/well scored on d 9; Exp. II, 100 cells/well scored on d 13; Exp. III, 500 cells/well scored on d 7). C: Clonogenic assay in SMMC-7721 cells. Cells were used in two experiments (Exp. I, 1,250 cells/well scored on d 5; Exp. II, 25,000 cells/well scored on d 5). D: SP population in SMMC-7721 cells was determined by Hoechst 33342 Efflux assay. **, p < 0.01 compared with SMMC-7721-pSuper and ##, p<0.01 compared with SMMC-7721-shCBX8.

**Figure S6. Overexpression of CBX8 could not reverse BMP4 knockdown induced proliferation and EMT inhibition.**

A: Proliferation in SMMC-7721 cells was examined by CCK-8 assay. B: Clonogenic assays in SMMC-7721 cells. C: Expression of epithelial (E-cadherin) and mesenchymal (N-cadherin, Vimentin, Slug, and Snail) markers were analyzed by western blotting. ** p < 0.01 compared with SMMC-7721-pSuper.
